# Supplementary material for: Virulence and Cross-Protection Conferred by an Attenuated Genotype I-Based Chimeric Japanese Encephalitis Virus Strain Harboring the E Protein of Genotype V in Mice
Source: Microbiol Spectr. 2022 Oct 27;10(6):e01990-22. doi: 10.1128/spectrum.01990-22 (PMC9769820; doi:10.1128/spectrum.01990-22)
Supplement: Supplemental file 1 — Supplemental material. Download spectrum.01990-22-s0001.pdf, PDF file, 1.2 MB [file spectrum.01990-22-s0001.pdf]

SUPPLEMENTAL MATERIAL

FIG S1 Levels of viral RNAemia and cytokine expression in blood

FIG S2 Levels of viral loads in brain of mice infected with JEV-GI/V mutants

Table S1 Neuroinvasiveness and neurovirulence test of JEV-GI/V in mice

Table S2 Virulence of JEV strains in mice

Table S3 Pathogenicity of JEV-GI/V mutants in mice

Table S4 $PRNT_{50}$ titers of sera from vaccinated mice against homologous and heterologous genotype viruses

Table S5 Information of JEV strains

Table S6 Primers used for construction of recombinant JEV

Table S7 Primers used for qRT-PCR

SUPPLEMENTAL FIGURE LEGENDS

FIG S1 Viral RNAemia and cytokine expression in blood. Mice (*n*= 4) were intraperitoneally infected with the indicated JEV-GI/V mutants at a dose of 10^3 PFU and blood samples were collected at 3 dpi for analysis of viral RNAemia and cytokine expression. (A) RNAemia was detected by qRT-PCR with primer specific to viral NS1 gene. (B) Fold change of IL-6 expression examined by qRT-PCR. (C) Fold change of IL-1β expression examined by qRT-PCR. (D) Fold change of TNF-α expression examined by qRT-PCR. (E) Fold change of IFN-β expression examined by qRT-PCR. (F)

Fold change of IFN-γ expression examined by qRT-PCR. A p value was generated by the Student's t-test. $^{**}p < 0.01$, $^{*}p < 0.05$.

FIG S2 Viral loads in brain of mice infected with JEV-GI/V mutants. Mice (n= 5) were intraperitoneally infected with the indicated JEV-GI/V mutants at a dose of 10^3 PFU and brain samples were collected at 7 dpi for analysis of viral loads. The viral loads were detected by qRT-PCR with primer specific to viral NS1 gene. A p value was generated by the Student's t-test. $^{***}p < 0.001$; $^{**}p < 0.01$, $^{*}p < 0.05$.

Fig. S1

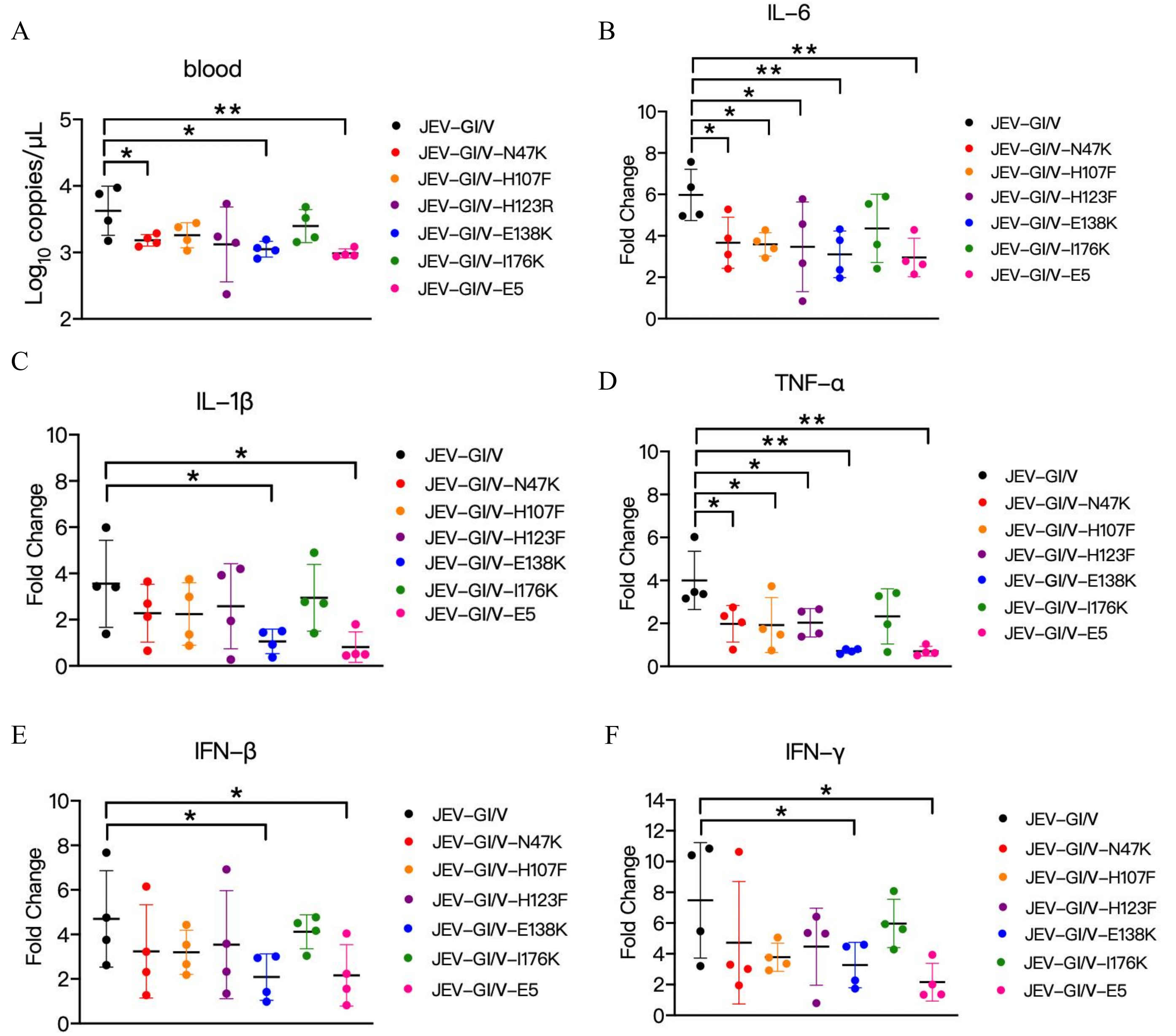
A
blood
Log10 coppies/μL
B
IL–6
Fold Change
C
IL–1β
Fold Change
D
TNF–α
Fold Change
E
IFN–β
Fold Change
F
IFN–γ
Fold Change
JEV–GI/V
JEV–GI/V–N47K
JEV–GI/V–H107F
JEV–GI/V–H123R
JEV–GI/V–H123F
JEV–GI/V–E138K
JEV–GI/V–I176K
JEV–GI/V–E5
*
**

Fig. S2

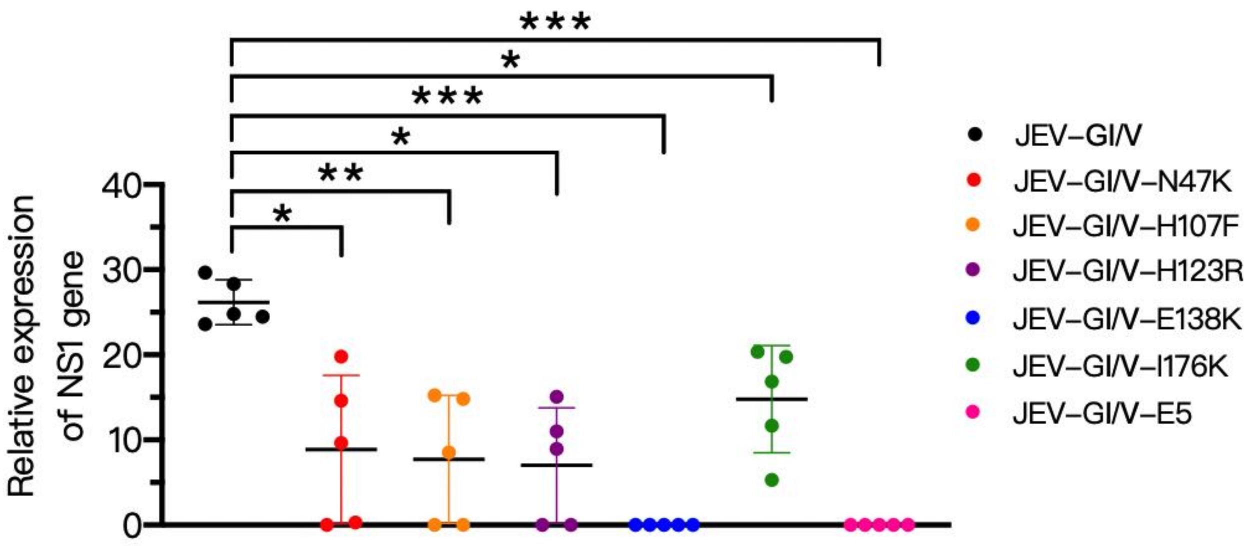

***
*
***
*
**
*
Relative expression of NS1 gene
40
30
20
10
0
JEV–GI/V
JEV–GI/V–N47K
JEV–GI/V–H107F
JEV–GI/V–H123R
JEV–GI/V–E138K
JEV–GI/V–I176K
JEV–GI/V–E5

TABLE S1 Neuroinvasiveness and neurovirulence test of JEV-GI/V in mice

Inoculation route	Dose (PFU)	Mortality (No. died/No. inoculated)	LD_{50} (PFU)
Intraperitoneal (neuroinvasiveness)	10^0	100% (8/8)	$10^{-1.5}$
	10^{-1}	87.5% (7/8)	
	10^{-2}	12.5% (1/8)	
	10^{-3}	0% (0/8)	
Intracerebral (neurovirulence)	10^0	100% (8/8)	$10^{-2.2}$
	10^{-1}	100% (8/8)	
	10^{-2}	62.5% (5/8)	
	10^{-3}	0% (0/8)	

TABLE S2 Virulence of JEV strains in mice

Genotype	Virus strain	Mouse strain	Mouse age	Virulence (LD_{50} PFU)		
				**Neuroinvasiveness**	**Neurovirulence**	**Reference**
Chimeric GI/V	JEV-GI/V (virulent)	C57BL/6	3 weeks	$10^{-1.5}$	$10^{-2.2}$	This study
Chimeric GI/V	JEV-GI/V-E5 (attenuated)	C57BL/6	3 weeks	$>10^{5}$	$>10^{4.65}$	This study
Chimeric GI/V	rJEV-$E^{XZ/SA14142m}$-M41 (attenuated)	ddY	3 weeks	$>10^{5}$	$>10^{3.48}$	Tajima al., 2020
GI	SD12 (virulent)	C57BL/6	3 weeks	$10^{2.7}$	$10^{0.4}$	Anwar et al., 2020
GI	SD12-F120 (attenuated)	C57BL/6	3 weeks	$>10^{6}$	$>10^{6}$	Anwar et al., 2020
GI	Mie/41/2002 (attenuated)	ddY	3 weeks	$10^{5.63}$	$10^{3.6}$	Nerome et al., 2007
GIII	N28 (virulent)	C57BL/6	3 weeks	10^{1}	$10^{0.7}$	Xiao et al., 2018
GIII	SA14 (virulent)	ICR	3 weeks	$<10^{0.18}$	$<10^{0.18}$	Yun et al., 2016
GIII	SA14-14-2 (attenuated)	ICR	3 weeks	$>10^{5.18}$	$>10^{5.18}$	Yun et al., 2016
GV	Muar (virulent)	ddY	3 weeks	$10^{2.5}$	-	Tajima al., 2015
GV	XZ0934 (virulent)	BALB/c	5-6 weeks	$10^{1.07}$	-	Cao et al., 2016

TABLE S3 Pathogenicity of JEV-GI/V mutants in mice

**Virus**	**Mortality at different doses (No. died/No. inoculated)**					
	**Intracerebral route**			**Intraperitoneal route**		
	**10^3 PFU**	**10^4 PFU**	**4.5×10^4 PFU**	**10^3 PFU**	**10^4 PFU**	**10^5 PFU**
JEV-GI/V	100% (8/8)	100% (8/8)	100% (8/8)	100% (8/8)	100% (8/8)	100% (8/8)
JEV-GI/V-N47K	100% (8/8)	100% (8/8)	100% (8/8)	62.5% (5/8)	100% (8/8)	100% (8/8)
JEV-GI/V-H107F	100% (8/8)	100% (8/8)	100% (8/8)	62.5% (5/8)	100% (8/8)	100% (8/8)
JEV-GI/V-H123R	100% (8/8)	100% (8/8)	100% (8/8)	62.5% (5/8)	100% (8/8)	100% (8/8)
JEV-GI/V-E138K	100% (8/8)	100% (8/8)	100% (8/8)	0% (0/8)	0% (0/8)	12.5% (1/8)
JEV-GI/V-I176R	100% (8/8)	100% (8/8)	100% (8/8)	100% (8/8)	100% (8/8)	100% (8/8)
JEV-GI/V-E5	0% (0/8)	37.5% (3/8)	50% (4/8)	0% (0/8)	0% (0/8)	0% (0/8)

TABLE S4 $PRNT_{50}$ titers of sera from vaccinated mice against homologous and heterologous genotype viruses

Group	Mouse No.	$PRNT_{50}$ titers against virulent viruses		
		JEV-GI/V	SD12(GI)	N28(GIII)
Mice vaccinated with JEV-GI/V-E5	1	60	5	30
	2	48	15	20
	3	52	12	16
	4	40	20	22
	5	56	5	24
	6	40	11	16
	7	56	10	22
	8	60	22	24
	9	42	18	18
	10	46	5	20
Mice vaccinated with SD12-F120(GI)	1	22	64	32
	2	5	68	22
	3	24	75	26
	4	22	62	5
	5	15	76	36
	6	5	60	20
	7	14	65	24
	8	20	70	5
	9	10	68	18
	10	5	56	28
Mice vaccinated with SA14-14-2(GIII)	1	18	20	50
	2	34	15	46
	3	20	28	44
	4	15	5	56
	5	24	22	58
	6	35	20	46
	7	20	22	62
	8	32	5	48
	9	28	27	50
	10	22	18	62

TABLE S5 Information of JEV strains

Genotype	Strains	Year	Country/region	Source	GenBank No.
GV	XZ0934	2009	China	*Culex tritaeniorhynchus*	JF915894.1
	Muar	1952	Malaysia	*Homo sapiens*	HM596272
	Tengah	1952	Singapore	*Homo sapiens*	KM677246.1
	K15P38	2015	South Korea	*Homo sapiens*	MK541529.1
	10-1827	2010	South Korea	*Culex tritaeniorhynchus*	JN587258.1
	K12YJ1203	2012	South Korea	*Culex orientalis*	KJ420592.1
	K12AS1148	2012	South Korea	*Culex pipiens*	KJ420590.1
	K12AS1151	2012	South Korea	*Culex orientalis*	KJ420591.1
	K12YJ1182	2012	South Korea	*Culex orientalis*	KM496505.1
	K12HC959	2012	South Korea	*Culex orientalis*	KJ420589.1
GI	SD12	2015	China	Pig	MH753127
	JS-1	2015	China	*Culex tritaeniorhynchus*	KX357114.1
	90VN70	1990	Viet Nam	*Homo sapiens*	HM228921.1
	SCYA201201	2012	China	Pig	KM658163.1
	HEN0701	2007	China	Pig	FJ495189.1
	K05GS	2005	South Korea	*Culex tritaeniorhynchus*	KR908702.1
	LN02-102	2002	China	*Culex modestus*	JF706278.1
	SXYC1523	2015	China	*Culex pipiens pallens*	KY078829.1
	SCMY	2014	China	Pig	KU351668.1
	YL2009-4	2009	Taiwan	*mosquito*	JF499789.1
GIII	SA-14	1954	China	Mosquito	M55506.1
	RP-9	1996	Taiwan	*Culex tritaeniorhynchus*	AF014161
	Beijing-1	1988	China	*Homo sapiens*	L48961.1
	N28	2015	China	Pig	MH753126
	K87P39	1987	South Korea	Mosquito	AY585242.1
	Fj02-29	2002	China	Pig	JF706273.1
	M28	1977	China	*Culex pseudovishnui*	KT957422.1

JH0418	2011	China	*Culex whitmorei and Anopheles sinensis*	JN381855.1
057434	2005	India	*Homo sapiens*	EF623988.1
YN	2011	China	*Homo sapiens*	JN381871.1

TABLE S6 Primers used for construction of recombinant JEV

Primer name	Primer sequence (5'-3')	Purpose
JEV-GI/V-1F	TACCTGACTCGAGTGTATAGTCGACAGAAGTTTATCTGTGTGAACTTCTT	Construction of infectious cDNA clone of JEV-GI/V
JEV-GI/V-1R	CTATGGAAAAACGGCTTTGGCGGCCGCTTGTGTGATCCAAGACATTCCCCCAAAG	
JEV-GI/V-2F	TACCTGACTCGAGTGTATAGTCGACCTCTTTGGGGGAATGTCTTGGATCACACAA	
JEV-GI/V-2R	CTATGGAAAAACGGCTTTGGCGGCCGCCCATGGAACACCGGGATCATCAATCAAGT	
JEV-GI/V-3F	TACCTGACTCGAGTGTATAGTCGACTTTCACTTGATTGATGATCCCGGTGTTCCA	
JEV-GI/V-3R	CTATGGAAAAACGGCTTTGGCGGCCGCGGCTTGTCAGCGTTCTTGATGAGAGTCCA	
JEV-GI/V-4F	TACCTGACTCGAGTGTATAGTCGACTGGACTCTCATCAAGAACGCTGACAAGCC	
JEV-GI/V-4R	CTATGGAAAAACGGCTTTGGCGGCCGCAGATCCTGTGTTCTTCCTCACCACCAG	
JEV-E47F	GACGTGCGCATGATAAAGATCGAGGCCACGGAA	Generation of JEV-GI/V-N47K
JEV-E47R	TTCCGTGGCCTCGATCTTTATCATGCGCACGTC	
JEV-E107F	GAAACGGATGTGGGTTCTTTGGGAAAGGCAGCA	Generation of JEV-GI/V-H107F
JEV-E107R	TGCTGCCTTTCCCAAAGAACCCACATCCGTTTC	
JEV-E123F	AGTTTGTCTGCAGCCGCAAGGCCATTGGAAAG	Generation of JEV-GI/V-H123R
JEV-E123R	CTTTCCAATGGCCTTGCGGCTGCAGACAAACT	
JEV-E138F	GAAAACATCAAATATAAAGTTGGAGTGTTTG	Generation of JEV-GI/V-E138K
JEV-E138R	CAAACACTCCAACTTTATATTTGATGTTTTC	
JEV-E176F	CCAATGCTCCTTCCAGAACTCTGAAGCTCGGA	Generation of JEV-GI/V-I176R
JEV-E176R	TCCGAGCTTCAGAGTTCTGGAAGGAGCATTGG	

TABLE S7 Primers used for qPCR

Primer name	Primer sequence (5'-3')
qJEV-NS1-F	GGGCCTTCTGGTGATGTTTC
qJEV-NS1-R	AAACCGCAGGAATVGTCAAT
qMouse-IL-6-F	ACCTGTCTATACCACTTC
qMouse-IL-6-R	GCATCATCGTTGTTCATA
qMouse-IL-1β-F	CGCAGCAGCACATCAACAAGAGC
qMouse-IL-1β-R	TGTCCTCATCCTGGAAGGTCCAC
qMouse-TNF- α -F	TCACTGGAGCCTCGAATGTC
qMouse-TNF- α -R	GTGAGGAAGGCTGTGCATTG
qMouse-IFN-β-F	AGTTACACTGCCTTTGCC
qMouse-IFN-β-R	GTTGAGGACATCTCCCAC
qMouse-IFN-γ-F	ATCTCTTTCTACCTCAGACTCTT
qMouse-IFN-γ-R	GTGTGATTCAATGACGCTTATG
qMouseGAPDH-F	TATGTCGTGGAGTCTACTGGT
qMouseGAPDH-R	GAGTTGTCATATTTCTCGT
